# Identity (Re)Construction of Female Adolescents with Substance Use Disorders

**DOI:** 10.3390/ijerph18137022

**Published:** 2021-06-30

**Authors:** Danielle Treiber, Lize A. E. Booysen

**Affiliations:** 1 Graduate School of Leadership and Change, Antioch University, Yellow Springs, OH 45387, USA; abooysen@antioch.edu; 2 Business School, University of Stellenbosch, Belville, Cape Town 7530, South Africa

**Keywords:** adolescence, identity development, substance use, grounded theory, situational analysis

## Abstract

Identity formation is a developmental milestone for adolescents, and their identities are constructed and re-constructed through their interactions with others and contextual factors in their environment. When considering adolescents with substance use disorders (SUD), often this developmental milestone is misappropriated, misunderstood, and misrepresented. The purpose of this article was to explore how adolescents with substance use disorders form identity and construct a sense of self. Firstly, we explored the identity formation and reconstruction of 20 female adolescents with SUDs based on an in-depth grounded theory methodology (GTM) which included a situational analysis (SA). Secondly, we offered a theoretical model to explain identity construction and reconstruction of adolescents with SUDs that emerged from this research. We conclude this article with practical implications for treatment, and care of adolescents with SUDs.

## 1. Introduction

Determining one’s identity is considered a developmental milestone for adolescents. In working to answer the critical question “Who am I?”, adolescents grapple with how their interaction with the world and the response from the world resonates with how they view and feel about themselves. Currently, many treatment modalities and therapeutic practices reinforce the purpose and need for attachment to the “druggie identity” [1]. The “druggie identity” refers to a false identity used by adolescents to achieve a sense of belonging through the use of substances. This research addresses how the “druggie identity” is reinforced through identity construction and reconstruction in multiple contexts. This research also addresses how the reinforced “druggie identity” creates a disconnected and skewed sense of the true self while also diminishing the concept of an intersectional self (the ability to have multiple integrated identities at once).

Understanding the role of the “druggie identity” using a holistic approach is a necessity in addressing the increasing number of adolescents diagnosed with SUDs, entering and re-entering treatment programs and the justice system, and prevention for those not already diagnosed. Unless the entire person is addressed while understanding the foundational role of the formed identity around substance use, therapeutic work may not have the impact intended and may actually support regression upon completion [1]. Overall, understanding the conditioning created by the “druggie identity” and certain treatment practices is essential in breaking the cycle that reinforces the necessity of this reputation and concept of self. The current study addresses how understanding the “druggie identity” and its impact on the adolescent can allow a break from a cycle that has the ability to keep one trapped.

### 1.1. Theoretical Foundation

Sensitizing concepts are those that are the background ideas that inform the overall research problem [2]. Theories around the interconnection of identity development, social context, and navigation of self influence the understanding and analysis of the data and thus are considered sensitizing concepts. In the following section, the theoretical foundation that framed the study and understanding of identity are presented.

### 1.2. Identity Formation in Adolescence

Erikson [3] proposed a psychosocial theory to account for human development, and Erikson’s [3] ego identity theory and the following empirical work by Marcia [4] are referenced most often when addressing identity development. Erikson [3] broadened the contemporary view of development to include social and cultural contexts. The conceptualization that both social and cultural contexts majorly influence the development of identity departed from the psychodynamic perspective on development that believed that one’s personality was established in childhood and never changed after that point [5]. Whereas there are eight stages in Erikson’s theory, the most relevant stage for this research is the stage of identity versus role confusion. Erikson [3] used this stage to characterize the powerful crossroad faced by adolescents in the transition to adulthood.

Adolescence is the time when youth face the major developmental task of forming a coherent sense of self [5]. Youth attempt to formulate identity through the process of asking “Who am I?” and “Who will I be when I grow up?” [6]. At this stage, research suggests that the quest for identity is initiated in part by biological and pubertal transformations [7], cognitive growth [8], and increased social awareness [9]. Kroger [10] explained that the major task of the adolescent period is to construct a personal identity. Schwartz [11] maintained that Erikson viewed identity as a continuum. This continuum ranged from synthesis to confusion. Synthesis is described as a set of self-determined ideals and confusion is the inability to derive a self- determined set of ideals [5,6]. The “ideal” identity is located somewhere in the middle of these two points: synthesis and confusion [12]. Individuals who successfully resolve this stage combine and integrate relevant earlier identification into a unique sense of self [5]. This successful resolution allows the individual to arrive at a sense of coherence within one’s identity. Those individuals who do not resolve this successfully remain in a state of confusion and are unwilling or unable to adhere to “a synthesized set of goals, values, and beliefs, and instead jumps from one set of commitments to the next” [5], p. 94.

Expansion on the literature surrounding identity status as defined by Erikson [3] and Marcia [4] has been considerable [5]. The empirical work connecting the causal link between identity and risk-taking behavior in adolescence needs to be established. Most of the work surrounding identity and externalizing problem behaviors is inconsistent and inconclusive [12]. This identity work has also focused on a specific identity status and whether it determines problem behaviors such as illicit drug use or problematic alcohol consumption. The current research attempts to address this gap by adding a more holistic perspective of identity construction in relation to substance use.

### 1.3. Identity Construction and Substance Use in Adolescence

The process of achieving a consolidated sense of self, necessary for psychosocial and relational adjustment [6], is incredibly difficult [10]. Kroger [10] asserted that one’s life pathways and decisions are guided by this consolidated sense of self. The range of ability for an adolescent to achieve this sense of self depends on a range of variables. The variables range from whether healthy attachments occurred with the youth’s primary caretakers during infantile development [5] to an immature prefrontal cortex leaving youth with increased impulsivity and risk-taking behavior with minimal inhibitory function [13,14]. These variables are complex and extend from individual to external macro system forces. Booysen argued that:

“Identity, or the question “Who am I?” includes not only “who I think I am,” and “what I think others think I am” (individual and collective identity), but also “how I act and who I become as a being” (relational identity). Identity can be viewed as a constant interplay between three levels of inclusiveness, individual identity, relational identity, and collective identity in a symbolic interactive way” [15], p. 4.

To understand the process of identity construction for an adolescent, the current research examined social identity theory [16,17,18], critical identity theory [18,19,20], narrative identity [21], identity work [18,22,23], discourse and identity construction [18,24,25], possible identities [26], and intersectionality [18,20,27]. Table 1 provides an overview and summary of these identity theories and their relation to adolescents with substance use disorders. Whereas intersectionality was used to frame identity understanding, it is embedded in critical identity theory and thus does not have its own column in Table 1. Intersectionality will also be addressed in depth in the following section.

In order to fully understand the concept of the “druggie identity,” it is necessary to understand that the individuals in this study were diagnosed with a substance use disorder according to DSM-5 criteria [28]. It is acknowledged that it is at this level of substance where the “druggie identity” becomes problematic as it is reinforced as a negative and primary identity. Using drugs is considered a risk behavior as it can compromise the psychosocial aspects of adolescent development [29]. Yet, it is important to acknowledge that all behavior cannot be seen as problematic per se, not all drug use becomes chronic [30,31,32], and experimental consumption is commonplace. In this sense, the approaches toward addressing substance use need to be holistic that avoid censorship and pathologization [29,32] and understand the progression of the “druggie identity.”

Currently, the dominant approaches toward addressing substance use include 12-step based therapy, therapeutic community, family-based interventions, behavioral therapy, cognitive behavioral therapy, motivational based therapy, and pharmacotherapy [33]. Treatment is often initiated by a formal enrollment into either an outpatient or inpatient treatment program [34]. Inpatient programs range from one to three months on average [35]. The specific length of treatment time is determined by recommendations of the program staff. Once inpatient treatment has ended, youth often return to their home environment, which can put them at higher risk for relapse [34]. A youth may also enter an outpatient program after finishing inpatient treatment or enter directly into outpatient programs without ever attending an inpatient program.

### 1.4. Intersectional Identity and the Stigmatization of Substance Use

Stigma is an insidious social force that has been associated with an endless number of attributes, circumstances, health conditions, and social groups [36]. Stigma encompasses behavior [37] and is a social process perpetrated by dominant groups to achieve goals of exclusion and conformity [36], which marginalized groups must navigate and contend with. Drug use is a characteristic that is contrary to a norm of a social unit where the norm is described as a shared belief that a person ought to behave in a certain way at a certain time [38]. Societal norms in the US cast drug use as an unacceptable behavior and, thus, many hold negative opinions about people who use drugs [39]. Illicit drug users are seen as weak, immoral, and as causing a risk to society [39]. Many of these perceptions are the result of political ideologies [40]. Stigma is also a key factor explaining mental health service disparities between groups [41].

Intersectionality highlights the ways in which groups experience marginalization [42]. Intersectionality allows the researcher to unearth the power and status embedded in identities, and show that by having intersecting identities, both opportunity and oppression are created [18]. Depending on the salience of a particular identity in a specific context, these intersecting identities can signal advantage, disadvantage, or both at the same time [43]. Cole [20] stated that intersectionality “requires that we think about social categories in terms of stratification brought through practices of individuals, institutions, and cultures rather than primarily as characteristics of individuals” (p. 445). Bowleg [44] claimed that researchers have the responsibility to connect participants’ experiences with sociohistorical inequality to explain how multiple identities intersect and interact with systems of domination. In relation to the “druggie identity,” this research explored the impact of the singularity and stigmatization of this identity in its sociohistorical context by exploring the lived experience of female adolescents with substance use disorders. By taking this vantage point, the researchers were able to unearth the importance of taking a holistic approach by first addressing the impact and influence of the “druggie identity” and secondly reintegrating identities that describe the intersectional individual.

The aim of this study was to uncover (i) the “lived experiences” of the processes and dynamics that exist within and around the adolescent with an SUD, and (ii) how these process and dynamics influences their identity formation. The main research question of this study was: How do adolescents with SUDs form identity and construct a sense of self?

## 2. Materials and Methods

In order to answer this question, a grounded theory methodology [45], including a situational analysis [46], was utilized to find out “all that was going on in this situation”. Grounded theory allowed the researcher to explore the systemic interactions between the adolescents with substance use disorders and others in their world [45]. Grounded theory also allowed the researcher to explore the interactions between the self and others and the system [45]. These interactions are directly connected to construction of identity and self-concept [3,4,5]. Specifically, grounded theory utilizes processes such as constant comparison, emergent analysis, and theoretical sampling, to pursue intersectional forces, power, and social dynamics [45].

In utilizing situational analysis, this study described the range of contextual influencers present that constitute “the situation(s)” of the study, and it explicated the coexisting and competing external forces within the environment that, if overlooked, could decontextualize the situation [45]. In this study, situational analysis identified and acknowledged the forces affecting the participants’ substance use disorder. It also highlights the existing relationships between and among these forces, and the potential influence they might have. Ultimately, situational analysis allowed the voice of adolescents diagnosed with substance use disorder in this sample to be heard within the complex context that contains it.

Participants. A purposeful sample of 20 females between the ages of 15 and 18, diagnosed with substance use disorders, were interviewed in this study (3 = 18 years old, 9 = 17 years old, 4 = 16 years old, 4 = 15 years old). All of the participants attended or graduated from a therapeutic boarding school that targets girls ages 13–17, in grades 9–12, involved in a variety of risky and/or addictive behaviors. The sobriety range of the participants was from 0 to 27 months. The length of sobriety was not related to the length of time at the therapeutic facility (i.e., 0 months of sobriety belonged to a graduate of the program).

### 2.1. Data Collection and Analysis

This study included three sources of data. Primary data were collected in 2018 and 2019 through semi-structured interviews with 20 participants, and 5 expert interviews. The third source of data were secondary data and included reviews of various relevant documentation. The data from the participant interviews were used in the GTM analysis to elucidate the social processes and contextual data that surround the lived experience of the participants. The findings from the participant interviews informed the data collection from the next two sources, the expert interview, and the document review. The situational analysis supplemented the GTM analysis and utilized the data from the expert interviews and documentation to look deeper into the social embeddedness of the participants’ lived experience within the co-existing competing forces in the context of drug abuse and dependency and treatment. NVivo 11, a computer-based program developed, owned, and trademarked by QSR International, was used to organize and categorize the data.

Participant In-depth Individual Interviews. Each individual participant participated in a semi-structured interview, which focused on interactions between the self and others and the system. Adjusted conversational interviewing was used for this study. The research design focused on setting up interviews purposefully, meaning that with the range of ages and the focus on understanding the developmental identity through those ages, interviews began with an individual in each age group. Questioning began with a preamble that laid the groundwork for understanding the experience of the interviewee. The preamble was followed by the interview question: If you were to tell a story about who you are, what would that story be? Interviews were recorded and transcribed and checked for accuracy by each participant. Over the time that interviews were being conducted, an iterative process of coding, memoing, and constant comparison occurred before and after the completion of each interview. A coding team was used to allow multiple perspectives on the meaning making of data. Interviewing happened until saturation was reached. In the context of interviewing, saturation refers to the point at which no new concepts emerge from the data. The creation of abstract and messy situational maps during the coding process of the participant interviews informs the focus of the expert interview and document review.

Expert Interviews. The 5 expert interviews focused on the forces that influence, compete, and co-exist in the environment of drug use, dependency, and treatment, and included interviews with experts from healthcare, therapy/treatment, criminal justice, integrative practices, and 12-step programs. The interviews were coded for general themes. The data and themes provided by these interviews were then compared with the data provided by the dimensional analysis. This comparison triangulated and expanded the understanding of the entire situation in context presented by the participants in phase I of the study. The information received from these interviews is elucidated by the findings of the situational analysis.

Document Review. The secondary data focusing on the contextual factors were used to supplement the interview data, and included reviewing relevant documents and media, such as articles, social media postings, and music lyrics, reviewing public media and articles, and diving deep into literature that would provide a fuller picture of the context. These documents were coded and analyzed in depth. The analysis of the discourse items was also compared to the data found in the expert interviews and dimensional analysis. Items used as discourse were determined by the information provided by all interviews and the analysis included coding for themes and constantly comparing to all information provided to understand the connection between all items in the entire situation.

Messy maps began the process to bring together the information from both the primary and secondary data discourses. Map making began early and was an iterative process where it shifted and changed based on data that comes in. All versions of the maps were copied and kept for reference in the analysis process.

### 2.2. Ethical Considerations

The population in this study was considered vulnerable because the participants were under the age of 18, and have been diagnosed with substance use disorder. Therefore, a full Institutional Review Board (IRB) application was submitted, which included discussion of the ethical considerations, study protocol, procedures, and techniques. The IRB application was approved prior to commencement of the study (approval #0249327).

Prior to each interview, the participants were required to give verbal consent to an approved informed consent form (ICF). Whereas parent permission was necessary for a minor to participate in the study, the ICF allowed the youth to be fully aware of their rights in the interview and provided consent. The process of reviewing the ICF was used to ensure each participant understood the full extent of the purpose of the interview, all possible risks associated with the study, and all steps taken to ensure confidentiality. Because these youths were vulnerable as a population, every step was taken to ensure their safety and autonomy. Each participant had access to mental health services after each interview.

A naming code was used for each participant and any identifiable material was removed from transcripts and in the reporting of data. In addition, as part of the informed consent, the youth was offered the ability to member check the transcript to ensure that it was a true reflection of the interview, and what they meant to say. Member checking is a validation technique used to ensure the trustworthiness, specifically the confirmability, of the research. In this technique, data were returned to the participants to check for accuracy and resonance with their experience. Member checking addresses the co-constructed nature of knowledge by allowing them the opportunity to ensure the data represent what they wanted them to represent.

## 3. Results

### 3.1. Findings of Dimensional Analysis

Dimensions are abstract concepts that are a component of the phenomenon under study [47]. While working to model dimensions in a way that represents the lived experience of the participants, core and primary dimensions were identified. Core dimensions are those that are unifying concepts that relate to all the primary dimensions [47]. The construction of an explanatory matrix allowed the researcher and research team to begin building a substantive theory [45]. Explanatory matrices allow the examination of each dimension in relation to the context, condition, process, and consequences of the situation [45].

Context indicates the boundaries for inquiry—that is the situation or environment in which dimensions are embedded. Conditions are the most salient of dimensions. Conditions are dimensions of a phenomenon that facilitate, block, or in some other way shape actions and/or interactions of the processes of a given phenomenon. Processes include intended or unintended actions or interactions that are impelled by specific conditions. Finally, consequences are the outcomes of these specific actions/interactions [47], p. 318.

The central theme (core dimension) that emerged from the data was *seeking belonging*. Participants filtered all experiences through this dimension. The five secondary themes (primary dimensions) were *shining the self; suffering; raising the red flag; disconnecting; and numbing the pain.* Table 2 presents a description and illustration of the themes (core and primary dimensions) that provide the central understandings of the lived experiences of adolescents with a substance use disorder. Table 2 also depicts the conditions, processes, and consequences of the dimensions, evidenced through a sample narrative.

From the explanatory matrices in Table 2, it is clear that the interactions between the participants, others, and the system culminate in a deep sense of seeking belonging that progresses over time in response to the components of each of the dimensions. Each particular section of the explanatory matrix, the conditions, processes, and consequences, interact and react to each other as the lived experience of adolescents with SUDs continues. This lived experience is also situated in a particular context that reinforces the systemic process. The following section provides the findings from the situational analysis that describes the entire situation and the contexts involved.

### 3.2. Findings of Situational Analysis

The themes (dimensions) discussed above are embedded in particular contexts of co-existing and competing forces which also play a critical role in the lived experience of the adolescent with SUDs. Four major contextual domains adolescents with SUDs need to navigate were identified: *the pursuit of happiness*, *addiction culture*, *treatment and therapy*, and *recovering self.*

The *pursuit of happiness* encompasses the contexts that determine the unrealistic expectations set upon the youth in the study. Those contexts include family systems, religion, gender, including gender roles and identity, and privilege (in the case of this specific sample). For example:

*So, I felt like I didn’t have a right to have those emotions. I felt like because I have all these things, I’m so privileged, I have all this stuff that I didn’t have the right to feel the way I did.* (Gabby, 16)

The macro-level system contextual forces in this domain include capitalism, popular culture, and American culture. Each of the forces provide a certain way of existing for these youth, providing a specific attitude, approach to life, a need to acquire wealth and possessions, and even determine the body type and beauty necessary to be successful and wanted:

*It was kind of more like the pattern from, not just my family, but the school that I was in, it was like nobody cared what you were doing or how you were doing it, so long as it looked good.* (Charlotte, 16)

The process of pursuing happiness determined in these contexts sets the youth up for seeking externally to themselves for what they feel they are missing, themselves.

Immersion in *addiction culture* encompasses the contexts that create the space and forces for the participants to be silenced and pushed away from institutions and self, and can be loosely subdivided into *addiction culture*, and *treatment and therapy*.

*Addiction culture* encompasses positions of contention where certain systems are debating between positions and those positions have major determinations as to what happens with these youth developmentally. Authority figures, policies, and approaches in the contexts of education, healthcare, and criminal justice create a polarity on decision-making around the well-being of youth based on bias and conflicting views around substance use. This domain perpetuates a deep lack of trust in adults:

*My other experiences with adults who—I guess I have two other experiences. Adults who work in schools, it’s their job to say that doing drugs is bad and all this stuff is bad so I don’t even know really their personal opinion. It is bad, but whether it’s something that they know it’s going to happen or I don’t know. I think that’s a problem because then who do kids go to when they need help. At least I didn’t know who I could go to about that.* (Trinity, 17)

It also creates a dehumanizing and othering experience for the young women in the context of federal and state governments and embedded War on Drug politics:

*I see it in the news too like with the opioid epidemic. Just adults saying this number of people does drugs. It’s very dehumanizing.* (Trinity, 17)

*Treatment and therapy* covers the contexts that determine accessibility, as well as the types of treatment or therapy available. This domain also encompasses positions of contention that allow for a continued immersion in addiction culture. Specific contexts include institutionalized treatment, 12-step programs, therapists, and government regulatory agencies. Each young woman was provided treatment differently, with different protocol, and with an original outcome of continued disconnection:

*The government actually, they called CPS because they were like, “Her parents are neglecting her. She is going to die if she keeps doing this.” So, they were like, “Okay, we’re going to put her in this prison ward.” So, they put me there for a long time.* (Lina, 15)

These young women were eventually able to attend a therapeutic boarding school due to the ability to access this type of support. Equitable access to meaningful, mental health support is also a context in this domain. The domains of *addiction culture* and *treatment and therapy* can be conflated in the larger context of *immersions in addiction culture*. The conditions that create *addiction culture* and the responses found in the domain *treatment and therapy* create a continuous immersion in a culture that reinforces the inability to change the narrative of the “druggie identity” whether someone is currently using substances or not. This ultimately creates an experience that does not allow an ability to move oneself from the “druggie identity” even if that is what is desired.

*Recovering self* encompasses the contexts that are in place to provide help and assistance to these adolescents through the relational pieces contained by the *therapeutic milieu*, as well as the processes that allow a reintegration of the shattered pieces of self. This domain is presented as the silenced voices and actors in the discussions of the domains *addiction culture* and *treatment and therapy*. The context of this domain includes integrative models and approaches to treatment and therapy, trauma-based work, and mindfulness-based mind–body practices. The contexts of this domain allow the youth to discover what the world is to them and who they are to the world on their own terms:

So, kind of just figuring out the world for myself. It was very hard working just not in the right directions. Now, it’s going to be in the right direction. (Toby, 18)

## 4. Discussion

**Figure 1:**
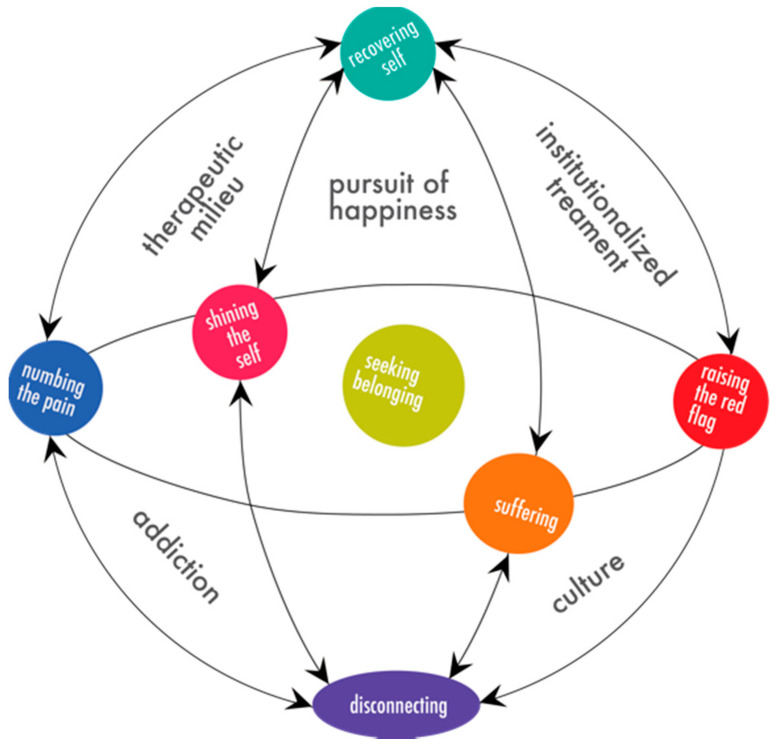
Theoretical model identity (re)construction of adolescents with SUDs.

The findings describe a complexly intertwined and systemic lived experience embedded in contextual and dynamic social processes involving these adolescents’ attempts at discovery of self. Figure 1, Identity (Re)Construction of Adolescents with Substance Use Disorders, is a conceptual model representing the dynamic processes and forces described in the data. Figure 1 shows how the identified themes and contextual domains relate and interact. This model is described in detail in order to show how the integration of the findings provide a full understanding of the contextual environment and systemic intra- and interpersonal interactions (lived experience) of adolescents with substance abuse disorders as reflected in the data.

The model presented in Figure 1 is a snapshot of this lived experience. Yet, there is nothing static about what the youth is experiencing. The experience ebbs and flows in and out of chaos, motion, and dysregulation. The youth is constantly using the most effective process in specific situations and domains that will lead to that core process, of *seeking belonging* (the green circle as the nexus of the central ellipsis in Figure 1), and the other primary processes orbit and inform the core process, *seeking belonging*. Note the arrows are double-sided, representing the movement from any part of the cycle to another, and the lines are not linear. Nothing about this process and experience occurs in a straight line, and these aspects are all intertwined and do not take place a linear fashion, and thus, cannot be treated as such.

The model is described in relation to the theoretical propositions in the following sections.

### 4.1. Theoretical Proposition 1: Development of the Pseudo-Identity through Substance Use Is an Adaptation to the Internal and External Environment of the Adolescent

The primary dimensions *shining the self* (the pink circle) and *suffering* (the orange circle) are situated closest to the core dimension, *seeking belonging*. There is a shift back and forth between these two dimensions and they emerge first in the social processes. As youth move into these processes, they begin to use their energy in creating a consolidated sense of self that aligns with the expectations set in the domain of the *pursuit of happiness*. As the youth shift into a version of themselves that is not true to the self, they fall into states of *suffering*, which creates a sense of dis-ease as the true self is shattering.

*Suffering* leads to trying to find new ways to please and appease through *shining the self*. The acceptance by others of a false version of the self defined by domain constructs leads back to *suffering*. It is in this process of developing the pseudo-identity, known as the “druggie identity,” where the first theoretical proposition emerges. The youth is using the “druggie identity” to maintain reputations that allow them acceptance and excellence. The external pressures from the *pursuit of happiness* have deemed these youth unworthy of their true selves and forced them into a false version that is actually accepted. Scholars across many disciplines speculate that a strong division of the world into a public sphere, where one conceals a stigmatized identity, and a private sphere, where one expresses that identity, becomes internalized in a form of an especially sharp distinction between public and private selves [48,49,50,51]. Self-schemas form around important aspects of the self and reflect domains of enduring salience, investment, and concern [52]. Self-silencing results through the process of self-suppression. This process is a relational schema whereby people suppress and hide affects, attitudes, and beliefs that might result in conflict with others, but are also predictive of greater depression [53] and psychopathology [54]. The false self leads to *suffering* and the “druggie identity” allows an avenue to create a new narrative.

The shift between *shining the self* and *suffering* is a continuous and arduous process. The two processes orbit around the core dimension *(seeking belonging)* and one pole is foregrounded while the other is backgrounded. This is relevant particularly in a therapeutic or treatment setting when the youth find the need to show how well they are doing therapeutically while hiding their *suffering*. By this time, the youth have mastered the ability to follow a program, manage a state of dis-ease, and place their *suffering* in the shadow.

### 4.2. Minor Theoretical Proposition 1: Behaviors Associated with the Pseudo-Identity Reflect the Adolescent’s View of Self

*Raising the red flag* (the red circle) is in response to *suffering*. It is the dimension when the youth allow their pain to be known. They ask for help. They act out. They self-harm. They attempt suicide. The youth begin to wear their wounds where they can be seen and hope someone will take notice.

The belief of Oyserman and Markus [55] is that the relationship between delinquency and the self-definitional task of adolescence may be reciprocal in nature and feeds into the cycle of delinquency and identity construction processes, where one follows the other due to their mutual influences on each other. The behavior is often considered anti-social, but the behavior reflects the way that the youth is attempting to communicate in relation to how they have come to understand themselves and the suffering associated with it. It is possible, in the scheme of delinquency, that youth who have a different level of social competence might find acts of delinquency as a way of trying on possible selves and determining which allows them to get positive feedback from their in-group, which reinforces their “druggy identity”, alienates themselves from possible other selves, and ultimately a consolidated self. According to Oyserman and Markus [55], if the youth have a lower level of social competence, they avoid adults and persons of authority and use peers for that feedback. The inability to attain that self results in impulsivity and increased vulnerability [55], which are risk factors for continued delinquency, as well as substance use, especially for adolescents.

The responses to the disclosure moments revealed in *raising the red flag* reinforce the lack of belonging and worth that the youth already feel in relation to not having their consolidated sense of self. In order for youth to actively and routinely disclose information, the relationship between adults and adolescents needs to be warm, accepting, and trusting [56]. This routine disclosure decreases when adults are psychologically controlling or react negatively to the disclosure [57]. What the youth learn in *raising the red flag* is that asking for help simply makes everything worse and reinforces self-hate. Eventually the responses in this dimension create loneliness which reinforces *suffering* and silences the youth which reinforces the need to *shine the self*, finally leading to *numbing the pain*.

*Numbing the pain* sits on the other side of the model. The positioning is counter to *raising the red flag*. If the youth are asking for help, they are not taking it into their own hands. It is when the youths do not get help or are not being taken seriously, that they decide that it is up to them to fix their problems. Promiscuity, popularity, and substances become a solution in this dimension. Entry into this dimension is not typically instigated by a desire to self-medicate, it is instigated by a peer or group of peers that is willing to accept the youth for a very minimal energetic exchange, such as drinking a beer, an attempt to achieve the consolidated sense of self. This dimension describes the youth’s version of identity work. Snow and Anderson [23] defined identity work as “the range of activities individuals engage in to create, present, and sustain personal identities that are congruent with and supportive of the self-concept” (p. 1348). According to Roberts and Creary [18], identity work can be considered the same as navigating the self. The core dimension of *seeking belonging* is at the center of this interaction, because group memberships speak to the psychological significance members derive from their membership, by fulfilling the need for self-enhancement, integration, and differentiation [15,18]. The youths are attempting now to find belonging within themselves through external means by identifying with the group, as the shattered self continues to create more dis-ease.

Once entry into this new friend groups occurs, the participants start to receive the attention they have been seeking for their entire lives. They find a feeling of belonging and derive their psychological significance from the group. Substances allow their anxiety to melt away and increase confidence, giving the youth a sense of power and control. They lose weight due to lack of self-care that is associated with substance use and thus, their bad body image does not seem as relevant when older men are contacting them and giving them attention. As the cycle continues, the participants continue to disconnect more and more from themselves, and integrate and assimilate more into the substance use group identity.

### 4.3. Theoretical Proposition 2: Core Cultural and Societal Positions around Treatment of Substance Use Have Direct and Indirect Effects on Well-Being and Identity Development of Adolescents

*Addiction culture* is the space where major cultural and societal debates exist. These debates create confusion around the understanding and treatment of substance use. In this space, the second theoretical proposition emerges.

Aspects of the self have been *disconnecting* (the purple ellipse) as the youth moves through the dimensions of *suffering, shining the self, numbing the pain*, and *raising the red flag*. The youth’s identity has been slowly shattering like a windshield that gets hit with a small pebble. The longer and bumpier the road, the farther the crack extends and more cracks appear, and the farther one becomes from a consolidated sense of self. Self-concept clarity is the degree to which self-knowledge is clearly defined, consistent, and stable across time and situations. Self-concept clarity is associated with the organization of self that is predictive of well-being [52].

It is through *disconnecting* that these youth are able to maintain the reputation of their pseudo-identity, the “druggie identity.” The youth are so disconnected from their true selves that they are afraid to stop their use. If they stop their use and attempt to reconnect the pieces of their shattering, the youth belief that there will be nothing of worth left in themselves. This stems from beginning interactions in *seeking belonging* and continued reinforcement through the social processes. The option is to continue use, disassociate from the pain of going against the self, and eventually become the void that is *disconnecting*. As can be seen in Figure 1, the domain of *addiction culture* pervades the space from the core and primary dimensions in the center down to the primary dimension of *disconnecting*.

Identity is highly personal and a social construction or culturally assigned social representation [58]. The specific content of these identities, such as what it means to be a “good adolescent”, differ with social and cultural context. Thus, “identity can be thought of as a social cognitive process and structure” [58], p. 201, and are produced by the “confluence of individual, relational, and collective level identities, negotiated in interactive dynamics, and meaning making and the sense giving processes embedded in socio-historical-political contexts” [15], p. 6. The specific socio-historical–political debates in this context present a conditioned zero-tolerant response that associate the “druggie identity” with that of the criminal, as a socially unacceptable person, and as “bad”. The youth experience these debates in schools, family systems, peer groups, religion, and treatment centers. These debates, in their simplest explanation, show the youth that they are not good enough to remain in the main population of their peers, there is something wrong with them, and that they need to keep their identity a secret. The reflection of the social context creates a deep sense of confusion that supports a shattered self vs. a consolidated self. This confusion and stigma leads to the extended energy and exhaustion around being seen as okay to others. Those with concealable stigmatized identities, such as substance use, who attempt to pass as “normal” are burdened with the preoccupation of concealing a secret in an attempt to hide their stigmatizing identity. This attempt to hide an identity causes a level of distraction by impression management and mental control tasks that they suffer in their performance in other cognitive tasks [59]. Higher levels of secrecy coping are related to lower levels of psychological flexibility, lower quality of life, more experiences of stigma-related rejection in the past, higher internalized shame, and most strongly with perceived stigma [60].

It is important to note that the *pursuit of happiness* provides the conditions for maintaining secrets through the culture of “no one talks about it”. Due to fear-based policies and treatments and the need to maintain a certain facade when existing in an affluent culture (as represented in this study’s sample), mental health, substance use, and addiction are not talked about. They are considered taboo, and the issues surrounding this are avoided. The youth are taught early on that talking about it either is going to get them labeled with diagnoses or the terms addict or alcoholic or it is going to deface the shiny status of their family and reputation, and that silence and avoidance are the best options. The youth finally give in, abandon any idea around getting to know themselves, and focus externally. Instead of being a person in context, they are a person as context embodied by the “druggie identity”. The balance between individuality and belonging are skewed toward belonging. The identity is sacrificed to the external while the youth give up on themselves.

At this stage in the process, death becomes the option to break this cycle. The concept around death can manifest as an overdose, suicide attempts, or suicide. In a state of complete disconnection, the youth believe their life will end soon no matter what they do and they accept that. They stop fighting. The desire to not experience their lives anymore is fueled by self-hate, acceptance of the pseudo-identity by the external world, and complete lack of energy allows the spiraling into deeper states of use.

### 4.4. Theoretical Proposition 3: Current Modalities of Treatment and Therapy, Specifically the Prescription of 12-Step Programs, Allows Reinforcement and Attachment to False Identities

As we move to the upper hemisphere of the model, a section that has not been addressed is the *therapeutic milieu*. At the bottom of that triangle are the primary dimensions of *numbing the pain* and *shining the self*. At the top of the triangle is the emergent property of the core dimension *recovering self* (green circle). *Therapeutic milieu* describes the relational pieces that draw these youth to find their *recovering self*. It is in pivotal relationships where finding the *recovering self* begins to be a possibility. The youth describe a multitude of people, peers and adults, who allow them to face the masks they maintain, address the pain underlying the numbing, and ultimately allow the shattered pieces of self to resurface so that these youth have the ability to pull them back together. Self-definition is always relational and comparative [17]. Throughout the youth’s life, healthy attachment to others and institutions was difficult to find. Adolescents with a higher sense of attachment or connection to their families and others displays lower rates of multiple problem behaviors [61].

Many of the participants describe that they continue their pattern of *shining the self* in therapeutic and treatment practices. Due to the deeply ingrained belief and understanding that they are privileged and getting therapy and being in treatment is a privilege due to limitations on access (specifically cost), the youth belief that they must be getting better since they are getting help. The youth will continue to put on the masks as treatment provides an extended vocabulary to create appropriate ones for the new context. They will find other more acceptable ways to express their addictive personalities (i.e., exercise) and they will feel the pressure of needing to find the self that will be accepted in this arena. Entrance into therapy and treatment still have the ability to maintain the effects of the immersion in *addiction culture.*

American society’s response to substance use has been reactive [61,62]. The systems to deliver treatment intervention are developed for the most common and costly substance use and allied psychological disorders once these problems have developed. Current modalities of treatment were designed for adults and based on the belief that zero-tolerance and abstinence is the only and best option, in other words those are the only alternative identities on offer. These modalities of treatment are not optimal for adolescents with SUDs [1,63], because it is focused on finding sobriety and eliminating drug use as the priority for healing the harms of substance use. This research indicates that the focus of treatment needs to be on supporting the development and integration of self, while healing the harms that are associated with being identified as a drug user and supporting the family system in re-defining their constructed narrative around the druggie identity. The historical, social, and personal context of the adolescents needs to be taken into account, because it can provide opportunities and constraints on the path to self-construction and self-discovery [64]. Self-discovery is an emotion-focused process [65]. The process is emotion-based because it involves one’s feelings or intuition to determine if a specific skill or activity resonates with the self.

In the context of the current treatment reality embedded in this research youth enter treatment and are asked to be sober. By being sober, they are letting go of the one version of self that they have mastered and found to be an integral identity in that it provides a level of acceptance and belonging. The youth then get diagnosed and often, 12-step programs are prescribed, which is more focused on sobriety, than self-discovery and development [66] and also provides a stigmatized identity (addict/alcoholic) that must be adopted in order to achieve belonging. The youth are provided options of which acceptable identities might fulfill the need to belong again instead of being given the skills and tools to uncover who they are. The youth then exist in a space where they are surrounded by multiple, conflicting definitions of who they might be, which does not align with who they truly are. Since the dominant current treatments and modalities used are still immersive in the *addiction culture*, and the identities on offer do not allow the youth to discover their true selves safely, their original adaptive use of the “druggie identity” is thus still maintained and is now perfected.

### 4.5. Theoretical Proposition 4: Integrative Practices Support Development of Relational Well-Being, Non-Attachment to Pseudo-Identity, and Reconnection to the Lost Pieces of the Self in Context

*Functional recovery*, the place where the youth find the liberated self in a reconstructed identity, is the option for breaking the cycle on this side of the sphere, and where acceptance of the druggie identity starts and other identities emerge. Self-discovery occurs during the process of shifting through identity alternatives and results in the discovery of one’s own set of unique talents, skills, and capabilities [6,65,67]. The youth in this study described that the person they were before and after drug use maintained the same values. These values were described as being used for different means depending on where they were in their process, yet they always existed.

*I think they’re the working version of my values from before. Because honestly, I think arrogance, for example, is just an extreme version of confidence. It’s just the nonworking version like before, the nonworking versions of my current values.* (Cassie, 15)

They also described losing core childlike characteristics.

*When I started caring what other people thought about me was when I kind of lost a lot of things that I had when I was little.* (Shannon, 16)

In order to reach *functional recovery*, the entirety of the self needs to be considered, reconstructed, and incorporated back into being. Processes such as mind–body practices, identity work, meditation, and play that allowed the youth to discover missing parts of the self and discover the one-ness of the self, allowed this to begin emerging. The youth take back all the pieces that they have handed out in all of their contexts and make their own decision on what those mean for them. They ultimately integrate the different converging and conflicting pieces of themselves, and take the leap to find the belonging within, as opposed to attaching to the belonging without. Well-being emerges when a system is integrated [68]. Integration can be defined as the linkage of separate elements into a functional whole, a process that is in an ever-moving state of being [69]. Well-being is a dynamic process that is in a continual state of emergence and it involves three elements: the mind, the brain, and relationships [69]. In moving into functional recovery and allowing the liberated self to emerge is where we find our final proposition.

This proposition also aligns with an intersectional lens on identity which proposes that “identities are not operating independently, but rather woven together and entangled in their relationships with each other with multiplying effects” and embedded in socio-historical–political contexts [15], pp. 14, 27.

## 5. Conclusions

Treatment of adolescents with substance use disorders needs to be unique to the soul needs of the youth, involve the youth, and be culturally and developmentally appropriate. By doing so, it is possible to adopt a realistic framework that allows the youth to live freely as an imperfect being and empowering them to take up their own agency. This research and the identity (re)construction model provide insight into the lived experience of these youth. By acknowledging the role of all forces at play in the etiology and treatment of SUDs at the micro, meso, and macro level, practitioners and organizations have the ability to shift the narrative of these youth to one full of hope, connection, and purpose. The developed pseudo-identity known as the “druggie identity” has profound negative implications for the success and overall well-being of these adolescents. Focus therefore should be in assisting youth towards de/re-constructing their identities to become fully functioning adults. Implications for practice and leadership are as follows:

Prevention–intervention efforts. Prevention and interventions efforts need to be developmentally appropriate, culturally relevant, and unconditional. Prevention efforts also need to begin as early as possible and not at the age of onset of substance use or in reaction to maladaptive tendencies. A sense of belonging needs to be fostered throughout the developmental trajectory across all contexts as well in order to foster effective prevention efforts. Taking and using a harm reduction approach allows for meeting individuals where they are and helps them achieve the goals they personally want to achieve.

Open, fearless conversations. An effort to create the means for these youth to have a safe adult and safe groups to have open and fearless conversations around considered taboo topics is necessary. These efforts can exist through using circles in classrooms, supporting family communication, addressing the undiscussables in systems, and educating and modeling how to have discourse often around difficult topics.

Reclaim and reframe identity. Providing identity-based intervention and prevention programs that allow youth to reclaim the need for socially based identities, including the “druggie identity.” In these types of interventions, using the understanding that this identity meets a universal human need allows a reframing of the identity and easier access to acceptance and integration. Programming that allows acceptance of the druggie identity also treats individuals with a sense of dignity that is deserving of all humans. Exploration of other possible identities open up new ways for youth to understand themselves, to integrate their identities and to be their whole selves. Integration using an intersectional and inclusive lens should guide the ways we approach development of any programming or curriculum.

Shared leadership. While an interconnected understanding of existence is powerful in reframing the understanding of ourselves, it needs to be mirrored in leadership practices. Collaboration across and within organizations and sectors is crucial in the work toward alleviating the strain of the polarity created by the debates around substance use. Shared leadership practices are recommended in all institutions that either involve or make decisions for youth of all ages.

Shared leadership practices should involve the youth at all levels of decision making, thus incorporating a removal of the typical top-down hierarchy seen in institutions. Student voice practices are supported by truly democratic relationships [70]. In these relationships, the youth are able to actively speak about their experience, share their opinion, and play an active role in the decisions made around their own treatment and the environment within which they exist. When incorporating these practices consistently and effectively, the youth develop a sense of efficacy and agency, along with a sense of autonomy and responsibility, the real ability to stand on one’s own feet while speaking their truth.

Policy. This research supports efforts to use restorative justice practices in the education, therapeutic, and justice systems. It also supports the need to decriminalize and legally regulate substances in the effort to support unlearning and reframing the narrative that those who use substances are criminals and bad people.

Limitations of this research are in direct relation to the specific population and thus, the data is not generalizable to other populations. Future research hopes to expand the implications for this model beyond the specific population presented in this article. It is also recommended that research around substance use and dependence in our youth begins to focus on the impact of the narrative and the harmful identities reinforced by the cultural drug narrative and ways to alleviate such harms.

## Figures and Tables

**Table 1:** Self-identity theory summary.

Bases of Comparison	Social Identity Theory	Critical Identity Theory	Narrative Identity	Identity Work	Possible Identities
General Concept of Self/Identity	Both a person’s knowledge that he or she belongs to a social group or category, as well as how one feels about that belonging	Identities are multiple, shifting, competing, temporary, context-sensitive, and evolving manifestations of subjective meanings and experiences in the social worldSocioeconomic, institutional, cultural, and historical boundaries play a significant role in the categories within which an individual or group exist	Allows the individual to present a story that is a reflection of the person in social context and all the messiness that comes along with a constant reconstruction of identity based on that interaction with social context	Range of activities individuals engage in to create, present, and sustain personal identities that are congruent with and supportive of the self-concept	Future-oriented components of self-concept are the possible selves that we could become, would like to become, and are afraid we might become
Meaning-Making	Derive value or meaning of our own group, social comparison between groups occurs to categorize in-group and out-group and to identify with one’s own group	Context, social meaning, power disparities, and historical intergroup conflict affect the meaning making process of identity formation	Internalized and evolving story of the self that a person constructs to make sense and meaning out of his or her life	Tactic used by people to get a greater understanding of who they are	Selves validated by others will become part of one’s identity Partners in identity negotiation provide feedback on the self; affect sense of self being developed and congruency with the future self
Tactic to Achieve and Sustain Positive Sense of Self	Group memberships fulfill the need for self-enhancement, belongingness, and differentiation	Challenge the status and power relations that are a part of identity	No single narrative frame can possibly organize everyday social life, and thus selves are constantly revised through repeated narrative encounters	Work done is to ensure that the world around them sees the self that is consistent with how the individual sees him/herself	Future self provides a sense of potential and an interpretive lens for the individual’s life
Response to Threatened Identity	Social mobility, social creativity, and social competition	Critical identity theorists do not examine social threats and responses the way that social identity theorists do, but understand difference as always contextualized in power relations	People perform their narrative identities in accordance with particular social situations and in respect to specific discourse	Distancing, dispelling, living up to idealized images, and feigning indifference	People are motivated toward futures they believe they can attain and avoid futures out-group members might attain
Agency	Uses tactics to make self-enhancing comparisons between groups	Looks to determine root causes of marginalization, stigmatization, and discrimination	Ability to reflect gender and class divisions, as well as, the patterns of economic, political, and cultural hegemony	Refers to what the individual does in order to navigate the self in social context and allows individual to claim desired identities	Possible selves are essential for putting the self into action
In Relation to Adolescents with SUDs	Role of social comparison, integration, and differentiation is central to identity formation and determining the saliences of particular identities in specific contexts	Construct information that is useful in the struggle against, marginalization, suffering, and oppression; agency and liberation tactics are at play	Cognitive development sets the stage for narrative identity; adolescents with SUDs range in cognitive abilities and thus narrative reflects the ability to begin thinking about who one really is and who one wants to become	Allows adolescent to construct socially validated identity that reflects aspects central to one’s sense of self; identity claiming and re-negotiation is important	Adolescents will act either congruently with the future self or refrain from becoming congruent with a future self that is not wanted; peer groups and attachment essential in future self-definition

Note. From Tajfel and Turner (1979), Spears (2011), Roberts and Creary (2013), Alvesson et al. (2008), Cole (2009), McAdams (2011), Alvesson and Willmott (2002), and Oyserman and James (2011) [16,17,18,19,20,21,24,26].

**Table 2:** Explanatory matrices from dimensional analysis—primary and secondary themes.

Dimension	Conditions	Processes	Consequences	Sample Narrative Evidence
Core Dimension				
*Seeking Belonging*: The need to find belonging comes from the youth’s needs not being met, by being othered in institutions, and not meeting expectations set by social contexts; the process leads to a state of dis-ease with the self, followed by the state of the shattered self	Unrealistic expectations Being othered Developmental needs unmet	Shining the Self Suffering Raising the Red Flag Numbing the Pain Disconnecting	Dis-ease Shattered Self Recovering Self	*I thought that if I surround myself with those people, then I would become one of the top people again. But then I kind of got tired of trying so hard and not actually getting it that I stopped. That’s when I first started hanging out with the popular party people. It felt good for me because it wasn’t as much pressure*. (Lulu, 17)
Primary Dimensions				
*Shining the Self*: How the participants create the image externally that they do meet the expectations set before them	Popularity and perfection Family culture Feeling less than	Molding self Keeping secrets	Exhaustion Depression False perception of Self Best of the worst	*It’s like you see flowers and pretty, just happy contentment, but then there’s just so much behind that isn’t a part of the story or isn’t meant to be seen in the story. The person, the main character, is really strong and they put off this really tough kind of badass front and they’re likable and stuff, but there’s just so much that they’re not saying and there’s so much more*. (Cassie, 15)
*Suffering*: Occurs in reaction to the pain that has incurred in the participants’ lives and continues to incur through the many attempts to find belonging and achieve acceptance; this is a lonely process	Pain and loss Parenting styles Being different Bullying	Rebellion Experimenting Being the parent	Vulnerability to “bad” influences Anger and resentment Relapsing	*And so there was a lot of pain that I didn’t know about. I couldn’t identify it. It was just like I had pain so I had to cover it up. I didn’t know what it was about. I was very, very lonely without knowing and I needed help. But I didn’t want to admit that because that would make me weak.* (Cassie, 15)
*Raising the Red Flag*: Represents the attempts the participants make to ask for help; the attempts begin very explicitly with questions and disclosure moments and due to the reactions and treatment in those moments, the attempts to find help become more extreme and destructive	No one talks about it Intergenerational disconnect	Asking for help Externalizing problems Being institutionalized	Losing trust Feeling dehumanized Something wrong with me Exposure to other options	*Over the summer, I had told my parents that I needed to go to the mental hospital because I wanted to die. And my mom was like, “Okay, just sleep on it and we’ll take you in the morning.” And then I lied through the admission because I realized I didn’t really want to go to the mental hospital. I just wanted my parents to acknowledge me. Even then they didn’t*. (Lily, 15)
*Numbing the Pain*: The participants feel alone and have found new groups that accept them for simply participating in recreational use; this process allows the participants to continue being the false version of self that is accepted and avoid the true feelings that arise in the sober moments	Bad body image Self-hate	Promiscuity Using substances as a solution Escaping the current moment	Sexual assault Shame and guilt Older kid attention Downward spiral	*I just, at all cost, didn’t want to feel pain and sadness.* (Toby, 17)
*Disconnecting*: The participants let go of the pieces of themselves that are not good enough to the point where they become a void; their only identifiers are external to them, are the behaviors they participate in, or the people they are enmeshed with	Existential crisis Being unlovable Appeal of drug life Movement from family	Maintaining reputations Extreme relationships Not caring Disassociating	Becoming the void No concept of self Drugs controlling my life	*But pretty quickly after that, I was very suicidal all the time and I could not be relying on drugs. I also wasn’t eating, not taking care of myself. Then when I would go home for holidays and things, I was completely covered up and I just didn’t talk to my mom and I stayed in my room. As a result, I let people use me and I got myself into some pretty bad situations. I don’t know, I wanted to be like other people so badly, but I couldn’t because I already had a taste of this other life. I couldn’t stop. I just didn’t know what to do*. (Trinity, 17)

## Data Availability

Data supporting the results can be found in the original dissertation and all codes and interviews are held in NVivo.
